# Management and outcomes of capitellum fractures in adolescents: A case series and review of the literature

**DOI:** 10.1016/j.ijscr.2025.111295

**Published:** 2025-04-12

**Authors:** Yacine Zouirech, Abir Manni, Loubna Aqqaoui, Houda Oubejja, Sarah Hosni, Fouad Ettayebi

**Affiliations:** a Department of Pediatric Surgical Emergency, Ibn Sina University Hospital Center, Children's Hospital of Rabat, Morocco; b Department of Pediatric Orthopeadic Surgery "B", Ibn Sina University Hospital Center, Children's Hospital of Rabat, Morocco; c Mohammed V University, Faculty of Medicine and Pharmacy, Rabat, Morocco

**Keywords:** Capitellum fractures, Capitellar shear fracture, Adolescents, Elbow trauma, Pediatric elbow injuries

## Abstract

**Introduction and importance:**

Shear capitellum fractures (CFs) are rare, particularly in pediatric populations, and pose significant diagnostic challenges due to the high cartilaginous content of the developing elbow. These injuries often involve small osteochondral fragments that may escape detection on standard radiographs, requiring a high index of clinical suspicion. Timely and appropriate surgical management is crucial to prevent long-term complications. This case series presents the outcomes of four adolescents with displaced CFs treated by ORIF using Kirschner wires (K-wires) in three cases and a spongy screws in one case, highlighting the reliability of K-wires fixation, especially in resource-limited settings.

**Case presentation:**

We retrospectively reviewed four adolescents (3 boys, 1 girl; mean age: 13.5 years) treated for displaced CFs at our Pediatric Emergency Department between 2019 and 2024. Three injuries followed falls on an extended elbow and one on a flexed elbow. One case was associated with an ipsilateral elbow dislocation. Fractures were classified as Type I (two cases) and Type IV (two cases). ORIF was performed using K-wires (three cases) or a spongy screw (one case) via lateral (two cases) or posterolateral (two cases) approaches. Postoperative care included four weeks of immobilization in 90° flexion using a posterior brachio-antebrachial (BAB) splint, followed by progressive mobilization and physiotherapy in two cases.

At a mean follow-up of 18 months (range: 12–24), all patients achieved bone union within 6–8 weeks. K-wires were removed at six weeks and the screw at six months. Full pronation and flexion were regained, with only minor residual limitations in extension and supination in two patients. Functional outcomes were excellent, with a mean MEPI score of 100. No complications were observed, and all patients and their families expressed satisfaction with the results.

**Clinical discussion:**

CFs in adolescents are uncommon and frequently overlooked due to subtle radiological findings. When clinical suspicion is high, advanced imaging such as CT is invaluable for accurate diagnosis. ORIF via lateral or posterolateral approaches using K-wires or screws provides stable anatomical reduction and preserves joint function. K-wires fixation remains a practical and effective option, particularly in low-resource settings.

**Conclusion:**

Early diagnosis and surgical management are essential in treating displaced CFs in adolescents. This series supports the effectiveness of K-wires fixation in restoring joint integrity and achieving excellent functional outcomes, underscoring its value in resource-constrained environments.

## Highlights

• Capitellum fractures in adolescents are rare and pose diagnostic and management challenges.
• Four pediatric cases analyzed to assess presentations and surgical outcomes.
• CT scans aid accurate diagnosis and surgical planning in elbow injuries.
• ORIF via lateral approaches using Kirschner wires and screws is effective.
• All patients had good recovery with no major complications. Early diagnosis and adapted surgical strategies improve pediatric outcomes.

## 1 Introduction

Capitellum fractures (CFs) are uncommon injuries affecting both adults and children, accounting for approximately 6 % of distal humeral fractures and <1 % of all elbow fractures [1]. In pediatric patients, they predominantly occur during adolescence—typically around age 14—just before fusion of the elbow's ossification centers. Due to the cartilaginous nature of the distal humerus, such fractures are exceptionally rare in children under 12 years of age [2,3].

The pediatric elbow comprises six ossification centers, which fuse sequentially during adolescence—starting with the capitellum, trochlea, and olecranon—typically around age 14 [4]. Before complete fusion, traumatic forces may result in small osteochondral fragments that are difficult to detect on conventional radiographs.

Diagnosing CFs in this age group is challenging due to the complex anatomy and incomplete ossification of the elbow, often leading to missed or delayed diagnoses. These fractures are part of the so-called TRASH injuries “The Radiographic Appearance Seemed Harmless”, which includes subtle and easily overlooked elbow injuries such as lateral condyle fractures and radial head osteochondral lesions [5]. Advanced imaging techniques—particularly computed tomography (CT) or magnetic resonance imaging (MRI)—combined with clinical vigilance, are essential for timely and accurate diagnosis [6].

CFs typically result from axial loading of the distal humerus when the elbow is in extension or semiflexion, most commonly from a fall on an outstretched arm [7]. Depending on the elbow's position at the time of injury, fragments may displace anteriorly or posteriorly. These fractures may also occur in conjunction with posterolateral elbow dislocation or subluxation due to the impact of the radial head and coronoid process on the capitellum [7,8]. Although CFs can occur in isolation, associated injuries to the lateral column—such as fractures of the radial head or neck, olecranon, or lateral ligament avulsion—are not uncommon [9].

Two main classification systems are used to categorize CFs: the Bryan and Morrey classification (modified by McKee), and the Dubberley classification [1,10,11].

**Fig. 1:**
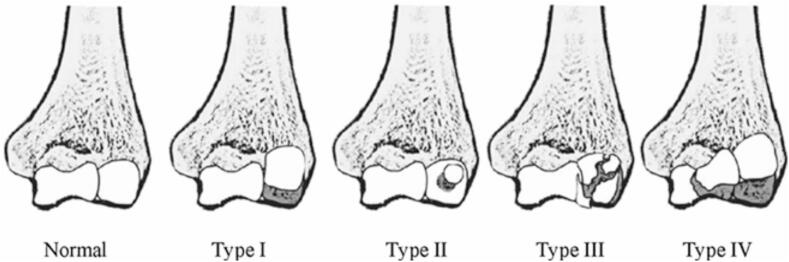
Bryan and Morrey classification, modified by McKee [1,10].
– Type I (Hahn-Steinthal): A large osteochondral fragment involving subchondral bone, sometimes extending into the lateral trochlear ridge.
– Type II (Kocher-Lorenz): A thin cartilaginous fragment.
– Type III (Broberg-Morrey): A comminuted version of Type I.
– Type IV (McKee): A fracture extending into the trochlear groove.
• Dubberley classification focuses on the extent of trochlear involvement and fragmentation, with subtypes A and B distinguishing the presence or absence of posterior comminution [11]:
– Type 1: Resembles a Hahn-Steinthal fracture and includes the lateral trochlear ridge.
– Type 2: Involves capitellar fragments extending into the trochlear groove.
– Type 3: Comminuted fractures involving at least two fragments—one from the capitellum and one from the trochlea.

• Bryan and Morrey classification includes four types based on fragment size and extent (Fig. 1) [1]:

Clinically, signs such as swelling, localized tenderness, hematoma, and restricted range of motion—often due to mechanical blockage or pain—can raise suspicion of a capitellum fracture. Radiographic indicators, including a double contour (suggestive of subchondral bone involvement) or a positive fat pad sign, should prompt further evaluation with advanced imaging. Given the anatomical complexity and variability in fracture patterns, CT is often essential when standard radiographs are inconclusive [2,7].

Management strategies depend on the degree of displacement and the specific fracture pattern. Displaced fractures typically require open reduction and internal fixation (ORIF), or fragment excision in the case of small fragments. In contrast, nondisplaced fractures may be managed conservatively with cast immobilization [2,3]. The final surgical approach is usually determined intraoperatively, with the goal of achieving anatomical reduction and preserving joint function.

## 2 Case presentation

We conducted a retrospective descriptive study of pediatric patients treated for displaced CFs at our Pediatric Emergency Department between January 2019 and December 2024. The study was approved by the local ethics committee. Inclusion criteria were: patients aged ≤16 years with radiologically confirmed displaced capitellum fractures, treated surgically by ORIF. Exclusion criteria included incomplete medical records, conservative treatment, or age > 16 years. We identified four cases displaced CFs described in this study and summarized in Table 1.

**Table 1:** Patient characteristics and outcome.

Case	Sex/age (years)	Mechanism	Diagnostic timing/imaging	Associated injuries	Treatment: ORIF	Follow up (months)	MEPI	ROM
1	13/M	Fall on the extended elbow while running	24 Hours/X-rays, CT scan	elbow dislocation	3 K wires	24	100	0°-150°
2	14/M	Fell on the extended elbow from a scooter	72 Hours/XR, CT scan	Any	3 K wires	18	100	15°-135°
3	14/M	Fall on extended elbow Fall from a bicycle	24 Hours/X-rays	Any	3 K wires	12	100	10°-140°
4	13/M	Fall on a flexed elbow	24 Hours/X-rays	Any	2 screws	18	100	0°-150°

Abbreviations: CT: Computed tomography scan. ORIF: Open reduction and internal fixation. MEPI: Mayo Elbow Performance Index. ROM: Range of motion.

Data were collected from patient medical records and included demographic information, mechanism of injury, imaging findings, fracture classification (Bryan and Morrey, McKee), surgical approach, fixation method, and postoperative care. Elbow pain, range of motion (ROM), stability, and function were evaluated using the Mayo Elbow Performance Index (MEPI) at final follow-up. Radiologic assessment of bone union was based on standard anteroposterior and lateral elbow radiographs. This study was conducted in accordance with the PROCESS 2023 criteria for surgical case series reporting.

Case 1 A 13-year-old right-hand dominant boy presented with pain, swelling, and limited elbow motion after falling on his outstretched left hand. Initial X-rays revealed an ipsilateral elbow dislocation with a posterior fracture fragment. A prompt closed reduction was performed in the emergency unit, and follow-up X-rays confirmed successful reduction but suggested a posterior CF. A subsequent CT scan confirmed a Type I (Hahn-Steinthal) posterior capitellar shear fracture with proximal and lateral displacement.

**Fig. 2:**
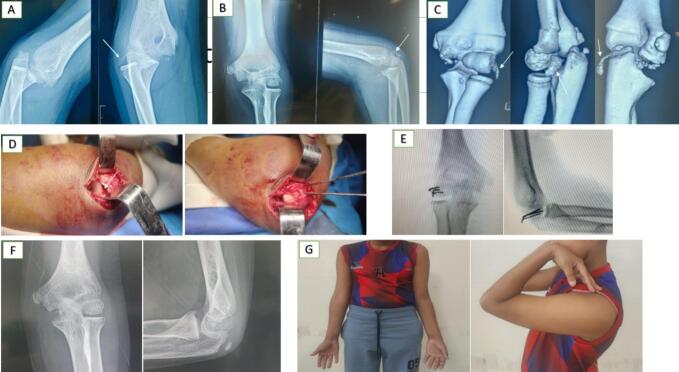
Case 1: Posterior Capitellar Shear Fracture (Type I - Hahn-Steinthal) with Proximal and Lateral Displacement Associated with Elbow Dislocation. (A) Anteroposterior and lateral radiographs at admission reveal elbow dislocation and a posterior fracture fragment (white arrow). (B) Post-reduction anteroposterior and lateral radiographs demonstrate successful reduction, with suspicion of a posterior capitellar shear fracture (white arrow). (C) 3D CT scan confirms the Type I (Hahn-Steinthal) capitellar shear fracture with proximal and lateral displacement (white arrows). (D) Intraoperative images depict the capitellar shear fracture and fixation using K-wires. (E) Postoperative anteroposterior and lateral radiographs show successful reduction and appropriate K-wire placement. (F) Follow-up radiographs at 2 months post-surgery confirm fracture union and anatomical alignment. (G) Clinical photographs at 2 months post-surgery demonstrate full range of motion (ROM) and functional recovery.

Surgery was performed the next day using a posterolateral approach. The fracture was reduced and fixed with three posterior-to-anterior K-wires. Postoperatively, the elbow was immobilized in a BAB half-cast for 4 weeks, followed by progressive mobilization with a hinged elbow brace. The K-wires were removed at 6 weeks, and radiographs at 8 weeks confirmed fracture union. The patient regained full ROM and resumed sports activities. Follow-ups at 6, 12, and 24 months demonstrated excellent functional outcomes without complications (Fig. 2).

Case 2 A 14-year-old boy presented with pain, swelling, and restricted elbow motion two days after falling from a scooter onto his outstretched left arm. Initially misdiagnosed by a local physician based on an anteroposterior plain radiograph, a lateral view at our department revealed a double contour sign. CT confirmed a Type IV (McKee) CF extending into the trochlear groove, with proximal and medial displacement.

**Fig. 3:**
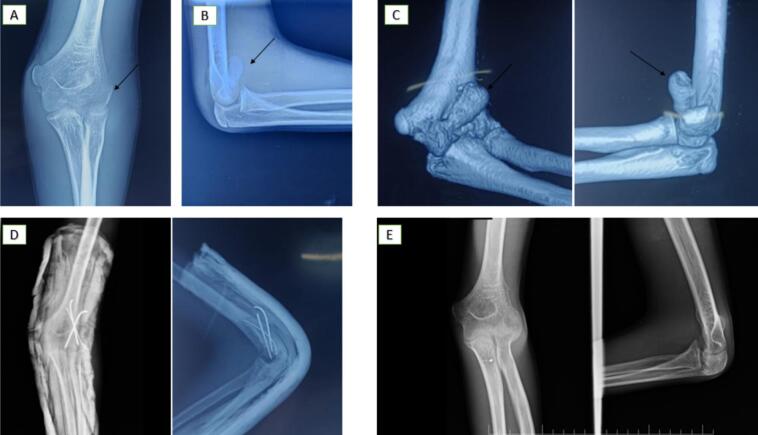
Case 2: Anterior Capitellar Shear Fracture (Type IV - McKee) with Proximal and Medial Displacement. (A) Anteroposterior plain radiograph showing a lateral fracture fragment initially missed (black arrows). (B) Lateral X-ray revealing a double contour sign. (C) 3D CT scan confirming the anterior capitellar shear fracture (Type IV - McKee) with proximal and medial displacement (black arrows). (D) Postoperative anteroposterior and lateral radiographs showing proper reduction and K-wire placement. (E) Follow-up radiographs at 6 months post-surgery confirming fracture union and anatomical alignment.

Surgery was performed the following day via a lateral approach. The fracture was reduced and stabilized using three K-wires left subcutaneously. Postoperatively, the elbow was immobilized at 90° flexion in a posterior half-cast for 4 weeks. After cast removal, a hinged elbow brace was introduced to assist mobilization. The K-wires were removed at 6 weeks, with radiographic union confirmed at the same time. The patient achieved near-full ROM (15°–140°), with only a minor extension deficit. Follow-ups at 6, 12, and 18 months revealed no complications, and the patient resumed all activities without limitation (Fig. 3).

Case 3 A 14-year-old right-hand dominant boy sustained trauma to his left elbow after falling from a bicycle onto his outstretched hand in a partially flexed and valgus position. Clinical examination revealed severe swelling, bruising, pain, and deformity, without neurological or vascular deficits. Radiographs demonstrated a fat pad sign and double contour sign, consistent with a Type IV (McKee) fracture involving the capitellum and trochlea, with anterior and lateral displacement.

**Fig. 4:**
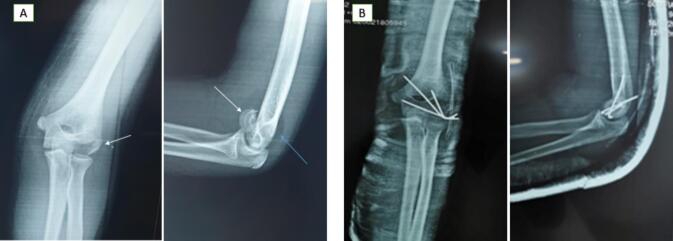
Case 3: Anterior Capitellar Shear Fracture (Type IV - McKee) with Proximal and Lateral Displacement. (A) Anteroposterior radiograph showing a lateral fracture fragment (black arrow). Lateral X-ray revealed a fat pad sign (blue arrow) and a double contour sign. (B) Postoperative anteroposterior and lateral radiographs demonstrating proper reduction and K-wire placement. (For interpretation of the references to colour in this figure legend, the reader is referred to the web version of this article.)

Surgery was performed within 24 h using a posterolateral approach, allowing clear visualization and accurate reduction of the displaced fragment. The fracture was stabilized with 3 divergent K-wires. Postoperatively, the elbow was immobilized at 90° flexion in a posterior splint for 4 weeks. Progressive mobilization was initiated after splint removal, and the K-wires were extracted at 6 weeks following confirmation of radiographic bone union. At 6 and 12-month follow-up evaluations, the patient demonstrated excellent recovery, with a ROM from 10° to 140°. Minor extension and supination deficits were noted compared to the contralateral elbow but were not associated with any functional limitations (Fig. 4).

Case 4 A 13-year-old right-handed girl sustained direct trauma to her right elbow during a basketball game. She presented with localized pain and swelling over the lateral elbow, with the joint locked at 90° flexion. Neurological and vascular examinations were unremarkable. Frontal X-rays revealed a fragment within the radio-humeral joint space, initially suspected to involve the lateral epicondyle. Lateral X-ray identified an intra-articular bony fragment with a double arc sign, consistent with a Type I (Hahn-Steinthal) CF with anterior and lateral displacement.

**Fig. 5:**
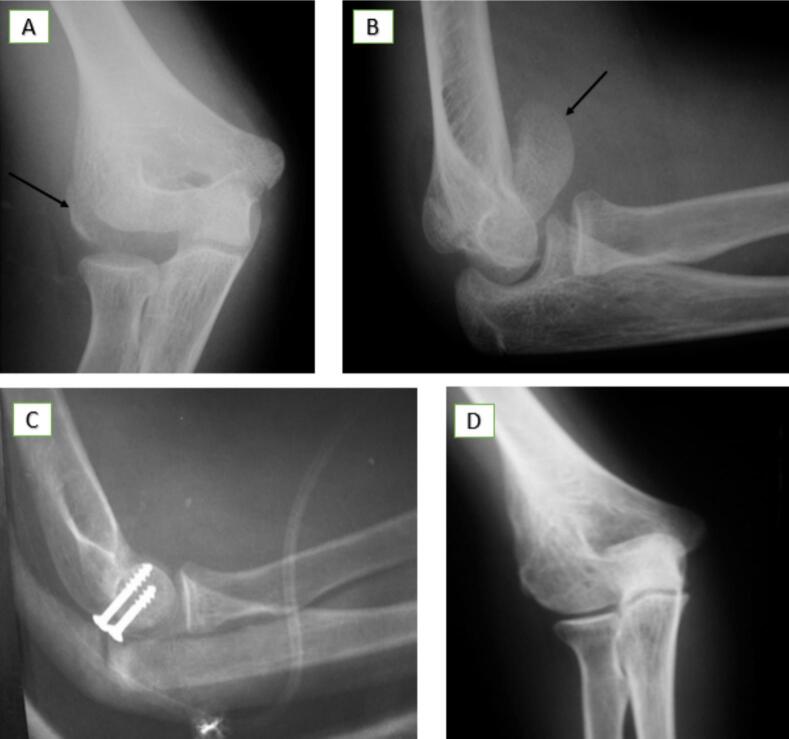
Case 4: Anterior Capitellar Shear Fracture (Type I - Hahn-Steinthal) with Proximal and Lateral Displacement. (A) Anteroposterior plain radiograph showing a lateral fracture fragment at the radio-humeral joint space (black arrow). (B) Lateral X-ray identifying an intra-articular bony fragment with a double arc sign, consistent with a Type I (Hahn-Steinthal) capitellar fracture with anterior and lateral displacement (black arrow). (C) Postoperative lateral radiograph showing proper reduction and placement of screws. (D) Follow-up lateral radiograph at 6 months confirming fracture union and anatomical alignment.

Surgery was performed within 24 h using a wide lateral approach, which provided optimal visualization for precise reduction. Stabilization was achieved with 2 posterior-to-anterior small spongy screws. Postoperatively, the elbow was immobilized at 90° flexion in a plaster splint for 4 weeks. Following splint removal, the patient underwent supervised self-rehabilitation, regaining full flexion and extension by the third postoperative month. At the 6-month follow-up, radiographs confirmed fracture union with proper alignment and no evidence of humeral condyle necrosis. The screws were removed at this time. By 18 months, the patient had resumed sports activities, demonstrating normal elbow function with no residual complications (Fig. 5).

## 3 Discussion

CFs are rare in the pediatric population, particularly before puberty [1,2,12]. Over a six-year period at our Pediatric Hospital, only four cases were documented, highlighting their low incidence and forming the basis for this study. Most published reports are isolated case descriptions or small series [2,12]. These fractures are predominantly observed in adolescents, coinciding with the progressive ossification of the distal humerus that renders the capitellum more susceptible to shear injuries [1,2,12]. In children under 12, the predominantly cartilaginous structure of the distal humerus typically redirects traumatic shear forces, leading to supracondylar or lateral condyle fractures, or small chondral or osteochondral fragments that are often undetected on radiographs [6]. This distinction underscores the changing biomechanics of the elbow with growth and skeletal development [1,2].

The typical mechanism of injury is axial loading on an extended or semiflexed elbow, as in a fall on an outstretched hand [2,7,12]. The capitellum's anterior location and its role as a contact point for the radial head make it particularly vulnerable to compressive forces, which may also cause associated injuries such as radial head fractures or elbow dislocations [2,8,11]. The most common mechanism is indirect trauma, such as a fall on an outstretched hand with the elbow in extension, which frequently displaces the fractured fragment superiorly and anteriorly [8]. In our series, three of the four cases were due to this indirect mechanism, with one case (Case 4) involving direct trauma to a flexed elbow, a less common but recognized pattern [12].

Fracture displacement direction is influenced by the elbow's position at the time of trauma. An extended elbow typically leads to anterior-superior displacement, while a flexed position can result in posterior displacement [2]. Additionally, CFs may be associated with posterolateral dislocation or subluxation caused by radial head and coronoid impact against the capitellum [2,7,8]. In our study, Case 1 presented with posterior displacement and elbow dislocation. The most frequent associated injury was the ipsilateral elbow dislocation, followed by the involvement of the medial or lateral condyle, or an olecranon or radial head fracture [2,11].

Radiographs, particularly in the lateral view, remain the first-line imaging modality for diagnosing CFs, especially when clinical suspicion is high. Classic indicators such as a “double contour” or a positive fat pad sign may suggest subchondral involvement [2,13]. While anteroposterior and lateral views, occasionally supplemented with oblique projections, are generally sufficient and cost-effective, their sensitivity remains limited—particularly in young children with unossified cartilage [2,5]. Historically, perioperative arthrography and oblique radiographs were employed to enhance fracture detection, but these have largely been replaced by more advanced imaging techniques [2]. CT and MRI now provide superior visualization of fracture morphology, especially in minimally displaced or complex injuries, and are highly valuable for preoperative planning [3,6]. In our series, CT was utilized in two cases to confirm the diagnosis and assess the extent and configuration of the fracture. Although standard radiographs were sufficient in the remaining cases, routine use of advanced imaging could improve diagnostic accuracy and guide appropriate surgical management, particularly in subtle or atypical presentations.

**Fig. 6:**
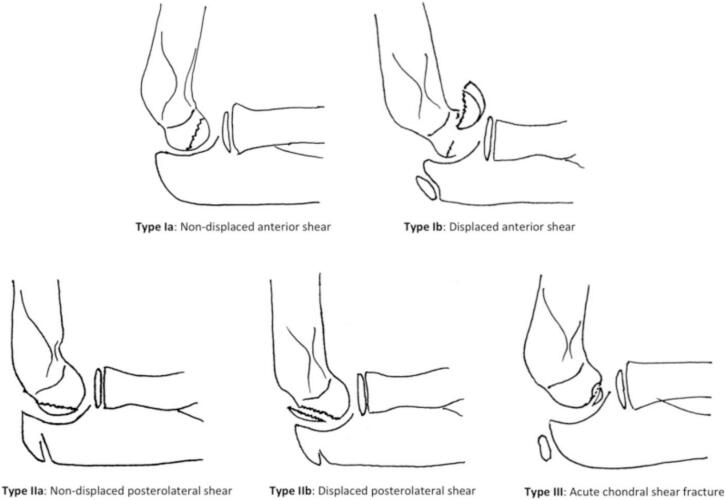
Classification of pediatric capitellar fractures as proposed by Murthy et al. [3].

Several classification systems aid in the categorization of CFs. The Bryan and Morrey system, modified by McKee, remains the most commonly used in pediatric cases [1,10]. The Dubberley classification, although more relevant in adults, focuses on the extent of trochlear involvement and fragmentation [11]. Murthy et al. recently introduced a sagittal-based classification specifically adapted for pediatric patients, which may offer improved surgical relevance in this population [3].This classification categorizes fractures into three types (Fig. 6):

- Type I: Anterior shear fractures, further subdivided into nondisplaced and displaced.
- Type II: Posterolateral shear fractures, often associated with ulnohumeral dislocations (consistent with our Case 1).
- Type III: Acute chondral shear fractures [3].

These classifications are crucial for surgical planning, with coronal extension of the fracture and any associated injuries serving as key factors in selecting the appropriate surgical approach [9].

The rarity of CFs in the pediatric population contributes to the lack of standardized treatment guidelines. Management strategies range from conservative approaches—such as cast immobilization with or without closed reduction—to surgical interventions including fragment excision and ORIF using implants like K-wires, Herbert screws, or cannulated cancellous screws [[1], [2], [3],^11^,^12^]. Prompt and appropriate intervention is essential to prevent complications such as joint stiffness, mechanical blockage, or long-term functional impairment.

Surgical approaches are selected based on the complexity and location of the fracture. The lateral approach is the most commonly used. The Kocher interval (between the anconeus and extensor carpi ulnaris) provides excellent access for posterolateral shear fractures [14], whereas the Kaplan interval (between the extensor carpi radialis brevis and extensor digitorum communis) is more suitable for anteriorly displaced fragments [9]. In complex cases, posterior (trans-olecranon), anterior, or anterolateral exposures may be required, while bilateral or extended approaches are reserved for fractures with medial extension or associated injuries [2]. Among these, the lateral approach remains the gold standard, offering safe and versatile access for both anterior and posterior lesions [2,3,12].

Common osteosynthesis devices include K-wires and screws. K-wires are typically left subcutaneously and removed within three to eight weeks postoperatively [2,13]. Headless compression screws, aligned with the sagittal plane, are increasingly favored for their mechanical stability and the advantage of not requiring removal [2,3]. Although not used in our series, bioabsorbable pins and osteosutures have shown encouraging results and deserve further investigation in future studies [15,16].

Small chondral or osteochondral fragments that cannot be securely fixed and result in mechanical blockage may require excision via open or arthroscopic techniques [3]. On the other hand, conservative treatment remains a valid option for non-displaced or minimally displaced fractures (<2 mm), typically involving four weeks of cast immobilization [3,12]. Surgical intervention is primarily indicated for symptomatic displaced fractures, with the most critical factor being mechanical blockage of the elbow's ROM [2].

Although our case series focused exclusively on displaced fractures treated with ORIF, we achieved excellent radiologic and functional outcomes in all patients, supporting the effectiveness of early surgical intervention in such cases.

The timing of diagnosis plays a crucial role in the management of CFs. Diagnosis is typically categorized as acute, when identified shortly after the trauma, or delayed, when recognized beyond two weeks post-injury [2]. Promptly diagnosed fractures are usually more significantly displaced and are readily identified on standard radiographs. These cases are typically managed with ORIF, which facilitates anatomical alignment and stability. In contrast, delayed diagnoses may necessitate a broader spectrum of treatment options, including conservative management, fragment excision, or late fixation, depending on the degree of displacement and the presence of mechanical symptoms [2,3,12]. In such cases, the choice of intervention is largely guided by the patient's clinical presentation, functional limitation, and the feasibility of achieving joint congruity.

Most patients achieve favorable clinical and radiological outcomes when appropriately treated [2,3]. Nevertheless, postoperative complications such as limited ROM, persistent pain, and joint blockage can occur [2,3,12]. Restricted elbow motion is often due to soft tissue contractures, which may necessitate arthrolysis, particularly when the functional arc is significantly compromised [3]. Conversely, joint pain and mechanical block are more commonly associated with radiocapitellar osteoarthritis, capitellar necrosis, intra-articular loose bodies, or chondral defects, all of which may require revisional surgical management [2,3,17].

Importantly, several studies suggest that complications such as osteoarthritis and osteonecrosis are not strongly correlated with the timing of diagnosis, as their occurrence appears similar in both early and delayed cases [2,3,12]. For instance, Mahirogullari et al. [17] reported osteonecrosis rates ranging from 0 % to 30 %, and Murthy et al. [3] observed symptomatic osteonecrosis in 24 % of patients. Malunion is rare but, when present, may require corrective osteotomy to restore joint function [18]. In our series, all patients were treated within 48 h of injury and none developed complications.

Our case series included four adolescents with displaced capitellum fractures (two Type I, two Type IV) treated by ORIF using K-wires (three cases) or spongy screws (one case), all achieving full functional and radiological recovery. The choice of osteosynthesis device should consider fracture type, surgeon expertise, and implant cost-effectiveness. Although our study did not compare outcomes between different fixation methods or surgical approaches, the results highlight the reliability of K-wires in restoring joint integrity, particularly in resource-limited settings. Future studies are needed to identify optimal fixation strategies tailored to fracture type and patient profile. Regardless of the approach or fixation method used, achieving gentle anatomical reduction and preserving the posterolateral vascular supply remain critical for favorable outcomes.

Based on our experience and a review of the literature, effective management of pediatric CFs starts with clinical and radiological suspicion. Lateral or oblique radiographs can reveal key signs, such as the double contour sign, joint effusion, or soft tissue edema. However, advanced imaging is often necessary for precise diagnosis and treatment planning. CT is the primary tool for confirming the diagnosis and assessing fragment displacement and size, essential for guiding surgical approach and fixation. MRI complements CT in younger children with a higher cartilaginous component or when evaluating associated injuries like ligamentous lesions.

Timely and accurate diagnosis is critical, especially for complex fracture patterns, such as displaced Type-II acute chondral injuries, which are often missed on standard radiographs. These injuries pose a high risk of complications, including nonunion, intraarticular loose bodies, impingement, and motion loss. The ultimate goal is to restore joint congruity and stability while preventing intraarticular fragments. Accurate diagnosis and early surgical intervention, when necessary, are key to achieving favorable outcomes and preserving joint function.

## 4 Conclusions

Capitellum fractures in adolescents, though rare and often misdiagnosed due to their subtle radiologic appearance, require early recognition and appropriate management to avoid long-term functional impairment. This case series reinforces the importance of maintaining a high index of suspicion and utilizing advanced imaging when necessary. Our experience demonstrates that open reduction and internal fixation, particularly with K-wires, is a reliable and accessible technique in achieving excellent outcomes, especially in resource-limited settings. Future comparative studies are needed to optimize treatment strategies according to fracture type and patient characteristics. Ultimately, precise anatomical reduction and preservation of vascular structures remain the cornerstone of successful surgical management in pediatric capitellar injuries.

## Research registration

Not applicable.

## CRediT authorship contribution statement

Yacine Zouirech: Conceptualization, Data collection, Writing – original draft, Writing – review & editing, Visualization.

Abir Manni: Conceptualization, Data collection, Writing – original draft, Writing – review & editing.

Loubna Aqqaoui: Methodology.

Sarah Hosni: Data collection, Supervision.

Houda Oubejja: Supervision.

Fouad Ettayebi: Supervision.

## Consent

Written informed consent was obtained from all patients or their guardians for inclusion in this study.

## Ethical approval

No ethical approval is required for de-identified patient records based on our institutional policies.

## Guarantor

Yacine Zouirech accepts full responsibility for the work, the conduct of the study, access to data, and the decision to publish.

## Sources of funding

No financial support was received for this study.

## Declaration of competing interest

The authors declare no competing interests or conflicts of interest related to this study.
